# Emotional expressiveness and avoidance in narratives of unaccompanied refugee minors

**DOI:** 10.3402/ejpt.v7.29163

**Published:** 2016-03-07

**Authors:** Julia Huemer, Kristin Nelson, Niranjan Karnik, Sabine Völkl-Kernstock, Stefan Seidel, Nina Ebner, Erika Ryst, Max Friedrich, Richard J. Shaw, Cassey Realubit, Hans Steiner, Katrin Skala

**Affiliations:** 1 Department of Child and Adolescent Psychiatry, Medical University of Vienna, Vienna, Austria; 2 Social Sciences Division, The New School for Public Engagement, New York, USA; 3 Department of Psychiatry, Rush University Medical Center, Chicago, IL, USA; 4 Department of Community, Systems and Mental Health Nursing, College of Nursing, Rush University, Chicago, IL, USA; 5 Department of Neurology, Medical University of Vienna, Vienna, Austria; 6 Nevada Center for Excellence in Disabilities, College of Education, University of Nevada, Reno, NV, USA; 7 Division of Child Psychiatry and Human Development, Stanford University School of Medicine, Stanford, CA, USA; 8 Abbey Neuropsychology Clinic, Palo Alto, CA, USA

**Keywords:** Trauma, unaccompanied refugee minors, narration, psycholinguistics

## Abstract

**Objective:**

The aim of this study was to examine a cohort of unaccompanied refugee minors (URMs) by means of psycholinguistic methods in order to obtain a more subtle picture of their degree of traumatization.

**Methods:**

Twenty-eight participants were included in the Stress-Inducing Speech Task (SIST) consisting of a free association (FA) and a stress (STR) condition. Narratives were examined by means of (1) quantitative parameters (word count); (2) psycholinguistic variables (temporal junctures, TJs), narrative structure, referential activity (RA)—a measure of emotional expressivity; and (3) content analysis ratings.

**Results:**

Word count was significantly lower than in age-matched norms. In the FA condition, TJs were lower, but in the STR condition, rates were comparable. RA was significantly higher in both conditions. Content analysis ratings showed that the experiences described by these youths were potentially traumatic in nature.

**Conclusions:**

This pattern of narrative shows a mixture of fulfilling the task demand, while containing an emotionally charged narrative. Narrative structure was absent in the FA condition, but preserved in the STR condition, as URMs struggled with the description of non-normative events. This indicates that these youths have not yet emotionally dealt with and fully integrated their trauma experiences.

A previous study on the prevalence of a range of psychopathology among African unaccompanied refugee minors (URMs) in Austria—youth who are under 18 years of age, who have been separated from both parents, and who are not under the care or supervision of an adult (Huemer et al., 2011)—found that rates of posttraumatic stress disorder (PTSD) were lower than expected (Huemer et al., 2011). This might be explained by the fact that compared with age-matched norms, this cohort did show significantly elevated levels of repressive defensiveness, restraint, and denial of distress, referring to an automatic adaptive style which is related to an investment in others’ positive valuation and a tendency to avoid disturbing affect and cognitions. These results could be interpreted as reflecting a capacity for resilience in URMs. However, for the majority of subjects, resilience actually co-occurred with indicators of vulnerability. For example, the URMs showed elevated trait distress (i.e., negative emotionality). Furthermore, significant correlations between psychopathology subscales, as measured with a self-report instrument, and repressive personality dimensions were found, suggesting potential trigger points for both aggression and self-destructive behavior. These discrepancies between results on symptom and trait scales among URMs suggested the need to further examine the potential relationship between stress, traumatization, and repressive coping mechanisms.

Measures used to diagnose PTSD most commonly include either self-report measures or structured interviews. Both of these methods have significant limitations—self-report measures are subject to avoidance and defensive distortion while structured interviews are based on clinician judgment, which sometimes is difficult in highly defensive subjects who are not able to provide access to internal thoughts, feelings, and emotions. Both these methods however may lead to under-reporting of symptoms, especially in those who habitually deny psychological distress or who have cultural constraints on the expression of emotion (Hashemi et al., 2008).

One way to circumvent the under-reporting of symptoms is to assess automatic mental processes at a level that is not easily willfully controlled, that is, at the unconscious level using psycholinguistic measures of spontaneous speech. In one standardized speech task, the Stress-Inducing Speech Task (SIST), the speaker is invited to discuss a specific topic, such as the recall of a prior stressful event (Steiner, Ryst, Berkowitz, Gschwendt, & Koopman, 2002). The resultant recording is transcribed and later analyzed using either standardized objective rating measures or computerized programs that incorporate a standard lexicon or dictionary (Pennebaker, 1995; Shanahan, Qu, & Wiebe, 2006). This approach will be used in the present study; its methodological underpinnings are described in the following sections.

## Linguistic analysis of trauma narratives

There are several inherent methodological problems that limit the accurate and valid description of automatic mental processes that operate outside of the individual's awareness (Steiner, Araujo, & Koopman, 2001). Efforts to circumvent this issue have resulted in a growing interest in the use of narrative methods as an alternative method of psychological assessment. Nelson and Horowitz (2001) and Nelson, Moskowitz, and Steiner (2008) have developed such a method, based on linguist William Labov's widely adopted analysis of narrative structure (Labov, 1972; Labov, 1997; Labov & Waletzky, 1967). As stated by Labov, the *sine qua non* for a story in all languages is the temporal juncture (TJ), which can be grammatically defined as the separation between two past-tense independent clauses told in an order that mimics that of the recalled events (Labov & Waletzky, 1967). This temporally sequenced, past-tense grammar signals an unconscious shift in speakers away from the usual “here-and-now” speech used in the opening and closing story sections, into what linguists call the “story-now”: speech oriented to past events, used for the action forming the heart of the story (Fleischman, 1990). The shift to “story-now” grammar (measured by number of TJs) grammatically marks the cognitive state in speakers where the past takes precedence over the here-and-now, inviting the listener into what psycholinguist Clark (1996) describes as a “joint imagining.” Thus, Nelson and Horowitz (2001) and Nelson et al. (2008) theorize that the presence of a high proportion of “story-now” grammar (measured by number of TJs) in retold personal memories indicates that a subject is immersed in reliving, rather than merely describing, past events. Such immersion in the “story-now” reflects a willingness on the speaker's part to let past memories unfold in sequence, re-activating the original “drama” of past events as recalled (Nelson & Horowitz, 2001). At the same time, this sequential retelling within the larger story structure makes past events comprehensible to the speaker itself and to listeners (Clark, 1994; Labov, 1997).

Thus, narrative retelling of past distressing memories indicates both the speaker's lack of defensiveness toward past experience and some regulation of this experience via structure. In a prototypical narration of a memory, the beginning and end anchor speaker and listener to the present and the past is allowed to predominate only during the temporally sequenced middle. Emotional experience is both expressed and given form within narratives. Interestingly, studies looking at the number of TJs in transcribed narratives of traumatic experiences have found that the presence of posttraumatic stress symptoms closely correlates with higher numbers of TJs (Shaw et al., 2001). The number of TJs has also been found to be strongly and positively associated with referential activity (RA), a measure of connection of verbal memory with sensory, somatic, and emotional experience (Nelson et al., 2008).

## Referential activity

RA which is defined as the degree to which a speaker's words convey the speaker's non-verbal sensory and emotional experiences is another important method used in the analysis of trauma narratives (Nelson et al., 2008). RA describes the extent to which a given verbal representation of sensory, bodily, and emotional experience evokes a corresponding non-verbal experience in the listener or reader (Bucci, 1997; Shanahan et al., 2006). Bucci (1997) argued that when speech is vivid (meaning that it is rated as clear, detailed, concrete, and imagistic, as opposed to vague, abstract, or general), it indicates that the speaker is translating sensations and emotions into words. According to Bucci's theory, vivid speech indicates that the speaker's non-verbal and verbal systems *refer* to each other. Vivid speech indicates that the speaker is accessing the analogical world of sensory and emotional experiences and translating it into the digital symbolic code of language (Bucci, 1997, 2002). Speech that is high in RA is likely to evoke corresponding specific, concrete, and imagistic memories in the listener. Components of RA are described in Table 1. To summarize, RA reflects emotional expression as embedded in experience, the corresponding psychological constructs being negative and positive emotion and affect. Therefore, just as temporal ordering within story structure is a linguistic reflection of the unfolding of personal episodic memories in an organized way readily communicated to others, RA is similarly postulated to link emotion with expression in a way that allows affective interpersonal sharing.

**Table 1:** Components of referential activity

RA subscale	Definition
Specificity	Quantity of detail
Imagery	Degree to which language evokes imagery
Clarity	Organization and focus
Concreteness	Degree of reference to sensory and other bodily experiences

*Source*: From Bucci (2002).

A further advantage of RA is that it offers computerized methods of analyzing text in an objective manner. The Weighted Referential Activity Dictionary (WRAD) comprises a word list of 696 items covering approximately 85% of spoken language and is used for computer scoring of RA (Bucci & Maskit, 2005). The Discourse Attributes Analysis Program (DAAP) uses the WRAD to produce a continuous measure of RA by computing the mean WRAD score. The mean WRAD score corresponds to an RA score, with a mean WRAD score of 0 corresponding to an RA score of 5 based on a rating scale of 0–10.

## The relationship between linguistic narrative analysis and RA

Evidence supports a relationship between psycholinguistic analysis and RA (Nelson et al., 2008). Psycholinguistic analysis, which includes the numerical count of TJs, describes the temporal ordering of speech and provides an indicator for the degree of the speaker's narrative immersion in the memory of the trauma through a structural analysis of the individual's account. By contrast, RA provides a measure of the sensory and emotional content of the trauma narrative. Although there is some overlap between linguistic analysis and RA with some research suggesting a correlation of approximately 0.69 (Nelson et al., 2008), each method does contribute a separate component to the analysis of the narrative. As such, both linguistic analyses and RA may be considered as separate but complementary measures of narratives.

In URMs’ descriptions of traumatic events, we anticipate that both narrative and RA will be present. The description of a specific event receives emotional coloring as expressed in RA, while form or temporal order is obtained by the narrative structure.

## The structure of trauma narratives

The literature on trauma narratives is complex (O'Kearney & Perrott, 2006). Cognitive models suggest that traumatic memories in individuals with PTSD are well retained, not particularly well integrated, in autobiographical memory and have specific characteristics that include the dominance of sensory, perceptual, and emotional impressions (Smeets, Giesbrecht, Raymaekers, Shaw, & Merckelbach, 2010). This pattern of expression reflects the degree to which the retelling of the traumatic memory includes particular details about the traumatic event, which entails the use of a variety of the brain's representational, sensory, and subcortical emotional systems that were activated at the time of that experience (Rubin, Berntsen, & Bohni, 2008). Emotionally evocative language and greater somatosensory detail, both features that are captured by the concept of RA (Beaudreau, 2007), are also reported to be related to “peritraumatic dissociation and the ‘flashback’ quality of the memories” that occur in PTSD (O'Kearney & Perrott, 2006).

Other findings from research on trauma narratives focus on the degree to which individuals process their traumatic experiences (Rubin et al., 2008). There is convergent evidence that suggests that trauma disrupts the brain's ability to linguistically and logically organize experience. This lack of organization could be reflected in deficient narrative structure, as measured by content analysis ratings and counts of TJs.

Past studies of trauma narratives do not take into account the role of defensive processes, which by definition involve automatic deception of self and others (Steiner et al., 2001; Shaw et al., 2001). Including measures of defensive processes may thus clear up some of the complexity in trauma narrative characteristics.

We know that a high degree of defensiveness is indicative of a capacity for repressive adjustment while at the same time it is negatively associated with the degree of narrative immersion (Nelson et al., 2009). We have found such repressive adjustment in a cohort of URMs (Huemer et al., 2011). In such a sample, we expect to find discrepancies between (self-reported and observer-rated) symptoms on one hand and psycholinguistic (disavowed) indicators of trauma on the other hand.

As the sample described here was quite small and as most previous analyses in larger samples had moderate effect sizes, we decided on an exploratory analysis, employing proper caution and restraint in interpreting the results.

Individuals with a history of an extraordinary or traumatic event will produce a narrative of their experience in one of the following ways:

1. Individuals may be avoidant of the narrative task altogether, that is, they may not speak much about their experience at all. In general, 300 words are considered the minimum amount for an adequate linguistic analysis. Narratives shorter than this are considered suspect, particularly if the word count is less than 100 words.
2. Subjects may produce a narrative of adequate length although almost entirely devoid of any relevant emotions. In a sense, such individuals are just “going through the emotion of being compliant” but in effect avoid any deep expression or experience of negative emotions.
3. Individuals might also produce a narrative in a highly emotionally charged fashion without creating comprehensible form or temporal order. In this case, the individual presents as being overwhelmed by the raw emotion and shows neither emotional nor linguistic ability to process these emotions.
4. Finally, individuals may describe the traumatic events using a proper admixture of emotional expression and narrative form. Such narratives may be considered to be the most optimal option; they indicate that the individual has had an opportunity to emotionally process their experience and in doing so develop an adaptive and useful perspective. Individuals demonstrating this narrative pattern are expected to display the healthiest profiles on measures of overall psychopathology.

Based on their history of traumatization, the memories of URMs investigated in this study were assumed to be not well-integrated in their autobiographical memory. The total words spoken and the number of stories within speech, both measures of narrative form, should therefore be high (Shaw et al., 2001). On the contrary, as these particular youths tend to show very high levels of conscious self-restraint and automatic, unconscious, repressive coping (Huemer et al., 2013), we hypothesized that their repressive personality structures might result in restricted descriptions and speech samples.

Second, given the nature and severity of the URMs’ traumatic experiences, we hypothesized that subjects would display high levels of sensory, perceptual, and emotional impressions, reflected in the psycholinguistic measure of text vividness, RA, representing their unprocessed emotional experience related to their trauma experience (Smeets et al., 2010). This was based on our hypothesis that URMs would not have had the opportunity or capacity to fully address their trauma experiences and instead rely on the use of repressive adjustment mechanisms which have been shown to be associated with the avoidance of traumatic memory retrieval (Huemer et al., 2011).

## Methods

### Study sample

Forty-one URMs participated in the study. Inclusion criteria were: (1) female or male African refugee minors aged 15–18 years residing in Austria in residential accommodations for URMs; (2) being unaccompanied by parents or close relatives, and URMs who did not display sufficient knowledge of English language were excluded from the study (*n*=50); 35 participants were male, six were female. Subjects came from a number of African countries, including Gambia, Somalia, Nigeria, Kenya, Ghana, and Eritrea. The countries of origin differed widely in terms of their geographic and cultural background. After complete description of the study, subjects gave written informed consent to participate. Administrative consent was also obtained from representatives of each participating institution. The questionnaires were distributed by trained personnel, who also offered instructions and guidance throughout the assessment. Recruitment procedures have been described in more detail in a previous paper (Huemer et al., 2011).

### Methods of recruitment

The present study was approved by the Ethics Committee of the Medical University of Vienna. All residential centers under the responsibility of the Austrian Public Welfare System were invited to participate in the study. Eight of a total of 15 institutions were included in the study. Reasons for exclusion of sites included an absence of African URMs in the respective institution, language difficulties, or refusal of the administrator or all minors living in the accommodation to participate in the study. Sixty-four URMs were eligible to participate in the study and of these, 23 (36%) refused to take part or did not succeed in completing the questionnaires. The 64 eligible URMs represented 40% of the total number of URMs (including youth from countries other than Africa) residing in the residential settings included in the study. The adolescents were assessed on successive days at their residential accommodation. Out of the 41 participants, 28 agreed to take part in the speech task.

In case-control analyses, subjects who participated in the described speech task did not differ significantly with respect to gender, age, nationality, psychopathology, and personality traits from those that did not (Huemer, et al., 2011; Huemer et al., 2013).

### Control group

The control group was recruited during a course on the study of stress and coping in adolescents from high schools in the Californian Bay Area. We chose this control group to obtain samples of English native speakers. Subjects were asked to participate in described SIST during which affective and physiological indices of stress would be obtained. A total of 168 students (84%) returned parent consents at school and agreed to participate. This sample of convenience was representative of the local high school population: ages ranged from 12 to 19 (with the exception of one participant aged 12 years and another aged 19 years, all participants were aged between 14 and 18). The mean age was 16.0 years (SD=1.3), the modal age was 15, and 55% were girls. Their ethnic and socioeconomic status reflected the surrounding suburban communities, being predominantly middle class and white (71.4% were European American, 11.4% were African American, 7.1% were Asian, 7.1% were Hispanic, and 3.0% were “other”).

### Measurement and evaluation techniques

#### Speech task

Participants completed several questionnaires (Mini-International Neuropsychiatric Interview for children and adolescents, the Youth Self-Report, the UCLA PTSD Reaction Index and Facts About You)^1^ followed by a standardized protocol called the SIST (Steiner et al., 2002). Subjects were asked to spend 10 uninterrupted minutes speaking into a tape recorder in each of two counterbalanced conditions: talking about (1) “your most stressful life event” (Stress condition, STR) and (2) “anything that comes to mind” (Free association condition; FA). Assignment to the conditions (which condition was performed first) was randomized. Subjects completed the two speech tasks without assistance, speaking into a tape recorder in a private room in the presence of a researcher who recorded the speech samples (Steiner et al., 2002).

#### Psycholinguistic analysis

First, the number of TJs and the number of stories were determined by a rater who was blind to all subject variables and conditions, in order to evaluate the degree of specificity in autobiographical memory. To define the presence of a story, the well-accepted linguistic definition of William Labov (1972) was used. According to this, stories consist of an “Abstract” or summary of the story for the listener; the “Abstract” is then followed by an “Orientation” to the who, what, where, and when of the tale; this is followed by a “Complicating Action” section, which always includes simple past-tense sentences spoken in chronological order. Finally the story ends with a “Coda” which reverts to the present-tense discourse of the speaker/listener situation. At minimum, a story needs to have a Complicating Action section; that is, two past-tense sentences spoken in chronological order. Stories told about the same theme were each counted. The total number of stories was summed for each transcript.

To determine the number of TJs, all sentences within a story (as defined above) were listed in the order spoken, then judged whether or not it was a sequential sentence, by asking: “does this sentence refer to a past situation occurring at a chronologically later time than the situation referred to by sentence 1?” (i.e., would a reversal in their order as spoken change the original semantic interpretation of the events described?). If the answer was yes, one TJ was scored; if not, sentences 1 and 2 were treated as temporally “overlapping,” and zero temporal sequences were scored. Moving to the third sentence in the list, the same judgment was made: “Does this sentence refer to events or states happening chronologically later than those in sentence 2?” (in case of a sequential sentence); or (if sentence 2 was not judged as sequential), “Does sentence 3 refer to a situation happening chronologically later than the situations reported by the set of sentences 1 and 2?” Again, if yes, one TJ was counted, if no, it was not, thus judging the entire list of story sentences. The number of TJs were then summed for each interview (Nelson, Bein, Huemer, Ryst, & Steiner, 2009).

#### Content analysis

In addition, two independent raters coded the following content variables for each interview: (1) How many events are described within each text? (2) What was the most important event? (3) Is the most important event normative (expectable event in specific developmental phase) or non-normative (stressful, non-expectable)? These ratings were performed by two independent raters who reached a correlation coefficient of >0.6. Finally, (4) the normative or non-normative event was rated by using a Likert scale (1–5), to establish how stressful the event was.

#### RA analysis

RA measures were developed in cognitive psychology to evaluate the degree to which words convey the speaker's non-verbal experiences (Bucci, 1997; Shanahan et al., 2006). Based on the experimental work of Paivio and Kosslyn, Bucci (1997) evolved a “multiple code” theory stating that RA between the speaker's multimodal non-verbal episodic memory systems and the verbal system will make words more likely to evoke corresponding specific, concrete, and imagistic memories in the listener. The current computerized rating is the latest of several RA dictionaries derived empirically from a large corpus of texts rated by readers for clarity, specificity, concreteness, and imagery (Mergenthaler & Bucci, 1999). In the present study, RA was computed using the most recent WRAD (Bucci & Maskit, 2005). The WRAD is a word list of approximately 700 items. Each item in the WRAD has a weight between −1 and +1; an item with weight of +1 is used more frequently in high-RA speech, while an item with weight of −1 is used more frequently in low-RA speech. Scores of 0.50 above or below the 0 mark are considered extremely vivid (high-RA) and extremely non-vivid (low-RA language), respectively. The actual computation of mean WRAD scores for our transcribed excerpts was done by the Discourse Attribute Analysis Program (HDAAP05), which, among other functions, automatically compares each word in a text with the WRAD, uses the WRAD weights as RA scores for the words (words not in the WRAD are given a score of 0), and then computes the mean WRAD score.

### Statistical analysis

Data analysis was conducted using the Statistical Package for the Social Sciences (SPSS) Version 15. Bivariate calculations (Spearman's rho) were applied to assess correlations. The type I error level was chosen to be 0.05. Content analyses were conducted by two independent raters. The two independent raters reached a correlation coefficient of >0.6. Differences between means were calculated via independent sample *t*-tests.

To determine corrected RA, mean RA was multiplied by the correctional factor, which is obtained by dividing 1,000 by the respective word count.

## Results

In terms of word count, the URMs cohort showed significant differences in comparison to the adolescent norm population (Nelson et al., 2009; Steiner et al., 2002). Word count, in both FA and STR conditions, was significantly lower for the URMs cohort than for the general adolescent population (*p<*0.05) (see Table 2).

**Table 2:** Quantitative parameters: word count; correctional factor

Quantitative parameters	URMs	Norm population
FA		
Word count (SD)	753 (407)*	1,296 (460)
Correctional factor**	1.3	0.8
STR		
Word count (SD)	670 (475)*	1,426 (509)
Correctional factor**	1.5	0.7

FA=free association, STR=stress, SD=standard deviation, URMs=unaccompanied refugee minors.

* *p*<0.05.

** Correctional factor=1,000/word count.

As assumed, the story content of URMs’ narratives differed from the norm (Nelson et al., 2009). Raters found that the events told in the stories were significantly more stressful than among the norm population (*p*<0.05) (see Table 3). As expected, considering the stressful past and continuing insecure status of these youth, many URMs told stories of their traumatic wartime and flight experiences in the FA, as well as in the STR condition. In contradistinction to normal adolescents, their trauma narratives were not limited to the STR condition but spilled over into the FA condition.

**Table 3:** Clinician ratings: rating of events

Clinician ratings	URMs	Norm population
FA		
Rating of events (how stressful?): 1–5 (SD)	2.7 (1.2)*	1.4 (.8)
Corrected per 1,000 words**	3.6	1.1
STR		
Rating of events (how stressful?): 1–5 (SD)	3.9 (0.9)*	2.6 (1)
Corrected per 1,000 words**	5.8	1.8

FA=free association, STR=stress, SD=standard deviation, URMs=unaccompanied refugee minors.

* *p*<0.05.

** Corrected referential activity=mean referential activity×Correctional factor.

In the FA condition, average TJ rate (number of TJs per 1,000 words) was lower than that of the norm population; in contrast, in the STR condition, the TJ rates were comparable between the norm and the URMs sample (see Table 4). RA of the spoken words, in both conditions, was significantly higher than those of non-clinical adolescents (*p*<0.05). The number of URMs’ stories was lower than among the norm population in the FA condition, and comparable with the norm in the STR condition (Nelson et al., 2009).

**Table 4:** Psycholinguistic variables

Clinician ratings	URMs	Norm population
FA		
Number of TJs (SD)	4.9 (10.1)	13.8 (15.0)
Corrected per 1,000 words**	6.5	10.6
STR		
Number of TJs (SD)	9.8 (12.4)	19.9 (18.0)
Corrected per 1,000 words**	14.7	14.0
FA		
Referential Activity (SD)	0.4 (0.1)*	−0.02 (−0.023)
Corrected per 1,000 words**	0.6	0.0
STR		
Referential Activity (SD)	0.4 (0.1)*	−0.04 (−0.026)
Corrected per 1,000 words**	0.6	0.0
FA		
Number of stories (SD)	0.7 (.7)	2.6 (2.0)
Corrected per 1,000 words**	0.9	2.0
STR		
Number of stories (SD)	1.3 (1.4)	2.6 (1.9)
Corrected per 1,000 words**	1.9	1.9

FA=free association, STR=stress, SD=standard deviation, URMs=unaccompanied refugee minors.

* *p*<0.05.

** Corrected referential activity=mean referential activity×correctional factor.

In Table 5, we have given some examples for narrative style.

**Table 5:** Extracts from narratives of different UMF

Trauma experience	… *Somalia, absolutely, you can't, you can't live there, the situation there is so difficult and people are dying there every day …*
	… e*very day, you think, if you are going to die …*
	*… bad things happened like killing the people aimlessly, also my family, my father was killed from Somali gun men, also my three brothers were killed by Somali gun men, one gunmen sold me, (he said) you will be married otherwise I will kill you and I don't want to be married to this man so he killed all the people … I don't want it if you don't want it he said if you reject me … I only will kill you …*
Coping strategies	… *I get nervous, nervous very easily … you know, so I sometimes when I get nervous, I meet people, you know, so I can calm down you know cause life, life is not easy, you know, too much, too much, too much stress, you know, too much stress for me, yeah, I miss all my people because of because of what has happened to me, to my life, you know, yeah, that also gives me stress, you know, but, but I think I am okay as far as I am living I am okay that's why when I have stress I meet people …*.
	… *When I am sad, when I think of my parents, you know, (I am) looking at the TV and my heart is cool, you know, when I don't look TV, I play the music, my heart is, I am cool, you know …*.
	*… When I am in a bad mood I like to stay alone because when I don't stay alone I can easily I can easily make a problem fight with somebody or hurt somebody so when I am angry … so in that mood if you meet me you don't want to you don't want to disturb me yeah I can say any bad word to you know I didn't intend …*
Future perspective	*… I want you to help me I want you to help me … Austrian ministry … I want you to help me … Austrian government … I would like … to get a peace where I can live … I am not sure … what will happen in the future …*
	*… I like to live and learn something and go to school that's enough I like to have a good life, my future to become good, I like to learn, this is enough …*
	*… The law says it is not written in my papers to work so this is why I lost this job and now I need a job if and I want to stay in this country I want for me to pay taxes in Austria think that it can be possible so please I need help I need I want to make work and before I work I need to make school and so that I understand the language that is more important if I can't speak the language for me to stay in the country it will be difficult …*.

## Discussion

The aim of the present study was to analyze the narratives of a cohort of URMs who were repeatedly exposed to non-normative, potentially traumatic events. We suggested that the use of these measures would reveal subtle indicators of psychiatric traumatization which may be missed by more conventional symptom checklists, structured interviews, self-report instruments, and personality inventories, similar to work described by Hashemi et al. (2008). Data supporting the use of psycholinguistic measures are informed by the theory of emotional memory formation (Bower, 1981; Nelson, McEvoy, & Schreiber, 2004). This theory supports the original Freudian contention that in free speech it is possible to find significant emotional links between narrated events, and that in specific situations an individual will display these links despite conscious efforts to control the narrative material (Vaillant, 1994). In this study, we sought to show that objective standardized measures of linguistic analysis of trauma narratives may be used to investigate subtle but relevant psychological indicators not evident in simple symptom self-report.

Primarily, we assumed that the narratives of our subjects would present profiles similar to those reported in other trauma samples. Thus, the number of words and stories within each condition was expected to be high indicating that a story is being told, as we have shown that TJs, storytelling, and number of words correlate positively (Shaw et al., 2001; Smeets et al., 2010). Contrary to our expectations, the word count for both conditions, FA and STR, was significantly lower than among the general population. The number of stories told by URM youth within a speech sample (corrected for words) was lower than among non-clinical youths. The degree to which URM youth displayed immersion (TJ rate) within the STR condition was comparable with the non-clinical norm. This finding of reduced volubility may reflect the conscious and successful attempts of URMs to integrate their traumatizing memories into a coherent narrative. But stopping short of ultimate results, the number of words spoken is usually consciously controlled, while more subtle indicators of narration are not. Limiting one's narrations by limiting the number of words spoken has been found to correlate positively with avoidant defensiveness, which in turn has been very high in these subjects (Huemer et al., 2013). The assumption that these youth had been affected by extreme adversity was corroborated by the blind and independent observer ratings of the narrative material, which revealed that the events told in URMs’ stories were significantly more stressful than among the norm population.

Second, we assumed that URMs would display high levels of sensory, perceptual, and emotional expressivity, represented by the psycholinguistic measure of text vividness, RA, reflecting their unprocessed trauma affect. This assumption was supported as mean RA was significantly higher than the comparable level in transcripts of non-clinical adolescents.

The present findings reveal that these youth do not display all the features of narratives that are typical and prevalent in traumatized patients; rather, they show some, but not all, features of the truncated narratives about distressing events, reported by avoidant defensive people in previous research (Nelson & Horowitz, 2001). This implies that these youth are not fully exploring the events they are describing. Such a more detailed description of events, with appropriate but not excessive emotional coloring, is usually found in narratives that have been told many times, with events “crafted” as they were, implying that the speaker has come to integrate experience and emotion and has coped with what has been described. In our sample, this does not seem to be the case.

Our findings show an interesting contrast between the words spoken and the amounts of sensory–emotional information within these words, as reflected by mean WRAD scores that were elevated compared with those of non-clinical adolescents. The implication here is that in the background of narration, we find a high level of RA which usually indicates high levels of emotional charge. In addition, URMs were similar to the norms with respect to the TJ rate (corrected for words spoken) in the stress condition, but not in the FA condition. This might be due to the fact that STR and FA conditions have different demand characteristics. While subjects can avoid talking in the FA condition, the STR condition directly confronts the speaker with the demand to unveil a non-normative event.

It is possible that non-native speakers of English might be expected to produce a lower number of words; however, if this were the primary explanation for the lower word count, one would also expect that the number of stories produced by the refugees would be many fewer than those produced by the non-clinical native-speaking adolescents, which is not the case. We assume instead that this finding may indicate an attempt of self-regulation and resilience, in which conscious defensive attempts to limit the reliving of trauma coexist with implicit “leaking” of the specific, clear, sensory–emotional details of experiences of war, loss, and flight. The finding of high ratings of emotional loading with fewer words spoken when compared with a norm population indicates that these youth are not integrating their experiences but are rather holding them in abeyance.

### Limitations and strengths

The small sample size is the primary limitation of this study. Recruiting a large number of the transient URMs population requires more resources than were available for this study. An additional limitation includes the use of measures which have not been validated or normed in ethnically matched samples from the youth's home countries. In addition, the homogenous profile of these youths, representing very similar values of mean RA and only little variance, makes it hard to compare or find correlative aspects with other variables. Furthermore, the speakers were non-native youth. Finally, one other major limitation of this study concerns selection bias—given a relatively high drop-out rate even after informed consent had been obtained. These youth presumably represent a group with significant pathology, even if case-control did not reveal significant differences on measures of psychopathology or personality. In addition, drop-out rates were higher in youth with poor command of the English language.

However, our study has several considerable strengths. Narratives were rated blindly and reliably for inclusion in the study. The tools used in the psycholinguistic analyses were able to reveal subtle aspects of our subjects’ traumatic experiences with consistent internal relationships similar to those reported in previous studies. In terms of psycholinguistic variables, a very interesting picture of defensive treatment of traumatic experiences can be described. High word counts and construction of stories in this group also entailed repression of emotions: volubility was constricted and narratives not fully developed; yet, measures of the sensory and emotional expressivity revealed by the URMs’ brief stories were much higher than compared with norms. These results mirror findings we have previously reported (Huemer et al., 2013). While describing potentially traumatic events, these youth defend themselves against the conscious expression of negative emotions, while at the same time showing a degree of emotional arousal that is evident in the RA of their trauma narratives. Thus, in the assigned task of describing the traumatic aspects of their experiences, subjects demonstrated a protective ability to contain their emotions. However, their efforts at containment were only partially successful, as the heightened emotional charge of the transcripts indicated. Ideally, we would expect that these youth fully processed their trauma—this would be reflected in a more elaborate narrative structure that could contain the greater emotional intensity of their stories. Their failure to provide this suggests ongoing vulnerability to trauma triggers as we have found in the study of their personality traits (Huemer et al., 2013) which may place them at risk for decompensation. Our findings support the idea that most of these youth could benefit from continued support and treatment in a safe and reliable environment that would therapeutically address unresolved trauma.

## Conclusion

Our results suggest that the analysis of a patient's narrative may provide clinically relevant information in cohorts who are coping with overwhelming events by using mechanisms of emotional distancing and avoidance. The potential clinical applications of this method of analyses are broad, and suggest that looking for the characteristics of narration in traumatized patients may serve as an additional informative diagnostic and therapeutic tool.

## Footnotes

1 Data on these are reported in Huemer J, Karnik N, Voelkl-Kernstock S, Granditsch E, Plattner B, Friedrich M, and Steiner H. Psychopathology in African unaccompanied refugee minors in Austria. Child Psychiatry Hum Dev. 2011 Jun;42(3):307–19.
